# A Web-Based Recovery Program (ICUTogether) for Intensive Care Survivors: Protocol for a Randomized Controlled Trial

**DOI:** 10.2196/10935

**Published:** 2019-01-17

**Authors:** Beverley Ewens, Helen Myers, Lisa Whitehead, Karla Seaman, Deborah Sundin, Joyce Hendricks

**Affiliations:** 1 School of Nursing and Midwifery Edith Cowan University Joondalup Australia; 2 School of Nursing, Midwifery and Social Science CQUniveristy Australia Brisbane Australia

**Keywords:** intensive care, survivorship, survivor, recovery program

## Abstract

**Background:**

Those who experience a critical illness or condition requiring admission to an intensive care unit (ICU) frequently experience physical and psychological complications as a direct result of their critical illness or condition and ICU experience. Complications, if left untreated, can affect the quality of life of survivors and impact health care resources. Explorations of potential interventions to reduce the negative impact of an ICU experience have failed to establish an evidence-based intervention.

**Objective:**

The aim of this study is to evaluate the impact of a Web-based intensive care recovery program on the mental well-being of intensive care survivors and to determine if it is a cost-effective approach.

**Methods:**

In total, 162 patients that survived an ICU experience will be recruited and randomized into 1 of 2 groups. The intervention group will receive access to the Web-based intensive care recovery program, ICUTogether, 2 weeks after discharge (n=81), and the control group will receive usual care (n=81). Mental well-being will be measured using the Hospital Anxiety and Depression Scale, The Impact of Events Scale-Revised and the 5-level 5-dimension EuroQoL at 3 time points (2 weeks, 6 months, and 12 months post discharge). Family support will be measured using the Multidimensional Scale of Perceived Social Support at 3 time points. Analysis will be conducted on an intention-to-treat basis using regression modeling. Covariates will include baseline outcome measures, study allocation (intervention or control), age, gender, length of ICU stay, APACHE III score, level of family support, and hospital readmissions. Participants’ evaluation of the mobile website will be sought at 12 months postdischarge. A cost utility analysis conducted at 12 months from a societal perspective will consider costs incurred by individuals as well as health care providers.

**Results:**

Participant recruitment is currently underway. Recruitment is anticipated to be completed by December 2020.

**Conclusions:**

This study will evaluate a novel intervention in a group of ICU survivors. The findings from this study will inform a larger study and wider debate about an appropriate intervention in this population.

**International Registered Report Identifier (IRRID):**

PRR1-10.2196/10935

## Introduction

An intensive care unit (ICU) stay is a stressful, potentially traumatic period for survivors and their families. Admission to an ICU usually means that individuals have suffered a critical illness or condition that is a threat to their lives, and they are among the most critically ill and vulnerable patients in the hospital. It is thought that the complications of a critical illness or condition are related to not only the severity of the illness but also the ICU experience [1,2]. Intensive care survivors often struggle to return to their previous role in the family and to their preillness state of health owing to prolonged physical and neuropsychological disability [3-5].

Over 172,000 people were admitted to ICUs across Australia during 2014-2015. As survival rates from ICUs have increased over the last 30 years, there has been a concomitant rise in the number of survivors, increasing the number of people who may develop chronic illness as a direct result of their ICU experience [6,7]. It has become increasingly apparent that those who survive an ICU experience and are discharged home suffer myriad physical, cognitive, and mental health impairments as a direct result of their ICU experience and critical condition [8-10]. These impairments can persist over a long period of time [4,11]. Psychological complications have been estimated to be as high as 44% of survivors at hospital discharge [1] and in some populations, they have been noted to increase during the year following hospital discharge [12].

Despite awareness of the high risk of complications post ICU discharge, support to anticipate and address physical and psychological complications is not routinely offered through existing health care services. Many survivors report being unaware of what to expect during recovery and lack knowledge of what is normal and when they need to seek help [13]. The onus to seek help post discharge sits in the hands of the survivor, and it is unknown how many “suffer in silence,” unaware if what they are experiencing is a usual part of recovery.

The purpose of this study was to determine if a Web-based intensive care recovery program improves the mental health and well-being of ICU survivors. As a mobile website, availability of the program will be unrestricted, enabling participants to access it at anytime and anywhere via a smart device. The program will enable participants to access support, advice, and guidance during their recovery post discharge from ICU.

## Methods

### Design

**Figure 1:**
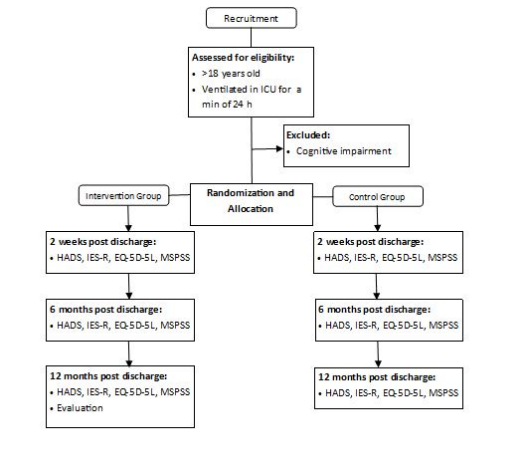
Study design. ICU: intensive care unit; HADS: Hospital Anxiety and Depression Scale; IES-R: Impact of Event Scale–Revised; EQ-5D-5L: 5-level 5-dimension EuroQoL; MSPSS: Multidimensional Scale of Perceived Social Support.

A parallel, prospective randomized controlled study will determine if a Web-based intensive care recovery program improves the mental health and well-being of ICU survivors. The study design for the protocol is outlined in a flow diagram in Figure 1.

### Ethical Considerations

Ethical approval was obtained from the participating study site and the university where the researchers are employed.

### Study Duration

Recruitment began in November 2018, and data collection will be completed within 2 years.

### Participants

Participants will be screened and recruited from a general 10-bed ICU within a 750-bed hospital in Metropolitan Western Australia following discharge from ICU and admission to a general ward. Eligibility criteria for Inclusion are as follows: aged 18 years and over at time of randomization, ventilation in ICU for a minimum period of 24 hours, able to speak and understand English, able to give informed consent, and access to an electronic device. Participants will be informed which group they have been randomized to immediately after providing written informed consent.

### Randomization

Permuted block randomization will be conducted in blocks of 20 using a computer random number generator. Allocation concealment will be conducted by an independent researcher using sequentially numbered, opaque, sealed envelopes. The independent researcher will provide the researcher conducting the consent process with the sequentially numbered, sealed, opaque envelopes. Envelopes containing the treatment allocation will only be opened by the recruiting researcher on participant enrollment. Blinding of the participant to allocation status will not be possible owing to the nature of the intervention. The researchers conducting the data analysis will be blinded.

### Intervention

Patients in the intervention group will receive access to a Web-based recovery program, ICUTogether that is accessible via smart devices and personal computers. The mobile website will provide information about health and well-being during recovery, including advice about exercise, sleep, and nutrition. Information will be provided on the recovery process, the signs and symptoms of potential complications during recovery, and when and how participants should seek professional help. Participants will be encouraged to keep a journal to promote reflection on progress over time and to explore their thoughts and feelings during their recovery. A chat room will also be available and participants will be able to post items to share with other participants in the study.

Participants will be given access to the mobile website 2 weeks following discharge from the hospital. A hard copy the self-help guide and telephone call will guide participants regarding the use of the site. If the participant is still unable to use the website, a researcher will visit their home and provide direct support with it. A demonstration of the functionality of the mobile website by a researcher will also be given at that time. The frequency of use of the website is at the discretion of the participants who will be encouraged to use the site as frequently as they wish. Each time a participant logs in, they will be prompted to complete a symptom checker that will identify material most useful to the participant at that point in time. The score from the symptom checker will be monitored and an alert notification will be sent to the researchers when the scores reach a certain level and thus gives cause for concern. Participants will receive a weekly email summarizing their participation over the previous 7 days and indicating any days they have not participated along with a prompt to continue participating.

The mobile website was developed by an external provider in collaboration with the researchers. The mobile website is being tested for ease of use and navigation properties by the researchers, clinicians, and a group of ICU survivors prior to commencement of the study.

### Control Group

The comparator, the control group, will receive usual care. Usual care is defined as ongoing management by the participants’ general practitioner (GP). There is no specific after care provision for ICU survivors offered at the study site. At the time of discharge from the hospital, survivors are discharged back to the care of their GP with additional services provided as necessary. These services do not include any specific ICU aftercare provision, for instance, follow-up by the ICU team. There will be no contact with the control group beyond consent and postal surveys.

### Data Collection and Measures

Following the consent process, demographic data will be collected from the participants’ health records, which will include the age, gender, admission diagnosis, severity of illness (APACHE III), existing comorbidities, and ICU length of stay. Data will be collected from participants at the following 3 time points: 2 weeks post discharge, 6 months post discharge, and 12 months post discharge (primary timepoint). The following three outcome measures will be used in the study: the Hospital Anxiety and Depression Scale (HADS), the Impact of Events Scale Revised, and the 5-level 5-dimension EuroQoL. All 3 measures will be completed at each of the 3 time points. The level of family support will also be collected at these time points via the Multidimensional Scale of Perceived Support [14]. Table 1 provides a brief description of each of the outcome measures. Participants in both groups will receive the 3 surveys by email 2 weeks post discharge from the hospital to complete the first data collection time point. At the following 2 data collection time points, the 3 surveys will be sent by via email to both groups. All participants will also be contacted by telephone to provide a prompt to complete the surveys at the data collection time points.

**Table 1:** Summary of outcome measures and description.

Outcome measure		Number of items	Description
**Primary outcome**			
	Hospital Anxiety and Depression Scale	14 on a 4-point scale	Used to identify the incidence of anxiety and depression in patients with a range of diseases and medical conditions [15].
**Secondary outcomes**			
	Impact of Events Scale Revised	24 on a 5-point scale	Measure of subjective distress in response to a traumatic event [16]. It comprises 3 subscales that represent the major symptom clusters of posttraumatic stress disorder: intrusion, avoidance, and hyperarousal [17].
	5-level 5-dimension EuroQoL	5 on a 5-point scale and a visual analog scale	Measure changes to health-related quality of life over time or between or following interventions [18]. The 5 dimensions within the survey are mobility, self-care, usual activities, pain or discomfort, and anxiety or depression.
	Multidimensional Scale of Perceived Social Support	12 on a 7-point Likert scale	Measures perceptions of support from 3 sources: family, friends, and significant other [14].

For participants in the intervention group at time point 3, 12 months after discharge, an evaluation will be conducted via the website on the acceptability and usability of the website. The evaluation will be conducted through the Web portal and completed online. The evaluation tool will be a modified version of the Mobile App Rating Scale [19] and comprise questions on the acceptability and content of the Web-based recovery program. The website is multidimensional, including recovery resources and an internet-based community, and facilitates journaling. To further understand the contribution each makes to outcomes, we will analyze the website analytics to determine how frequently and for how long the participants interacted with the site and the individual components within it. Participants will also be asked to rate each component of the site in terms of usefulness and perceived impact on their recovery.

### Sample Size

Sample size and statistical power were calculated for the primary outcome measure using G*Power 3.1.9.2 [20]. Detecting a 2-point difference (effect size of 0.44) between the 2 groups on HADS with an alpha of .05 and 80% power requires 81 participants in each group for a total sample size of 162 participants. Given the discharge rate of 80 patients per month and a recruitment rate of 30%, it is estimated that it will take 7 months to recruit the required sample size.

### Statistical Analysis

Data will be reported in accordance with the Consolidated Standards of Reporting Trial. Data analysis will be conducted using SPSS version 24 (IBM) and Stata SE version 15 (StataCorp). The characteristics of participants in each arm of the study will be summarized using descriptive statistics. Analysis of characteristics to determine similarity between the arms will be conducted using chi-square tests for categorical data and independent *t* tests for continuous data.

Further analysis will include stratifying the APACHE III scores into categories to determine if there is a difference in the level of severity and outcomes. The 3 outcome measures (HADS, IES, and QoL) at follow-up will be analyzed as dependent variables using regression modeling. Covariates will include baseline outcome measures, study allocation (intervention or control), age, gender, length of ICU stay, APACHE III score, and level of family support. Analysis will be conducted on an intention-to-treat basis. A subanalysis of the intervention group based on those who engaged with the mobile website compared with those who did not will also be conducted. The characteristics of people who withdraw from the study will be compared with those who remain in the study. Missing data will be addressed using multiple imputation assuming variables are missing at random. The person who completes the data analysis will be blind to participant allocation status. Summary statistics of frequency of use, time spent using the site, most frequently used elements, and time spent on each screen will be reported.

### Economic Outcomes

A cost utility analysis will be conducted at 12 months to determine whether the Web-based recovery program is a cost-effective approach for improving the quality of life of ICU survivors when compared with usual care. The perspective taken for the economic analysis will be a societal perspective and will include costs incurred by individuals as well as health care providers.

#### Identification of Costs and Benefits

The primary outcome measure for assessing benefits will be quality of life. The identification of costs will be across the following 4 main areas:

1. *Intervention costs* include ongoing mobile website maintenance such as hosting costs as well as the cost of promoting the mobile website to future ICU survivors. The cost for developing the mobile website and implementing the intervention for the study will not be included because these costs will not be incurred in future implementation of the mobile website.
2. *Health care costs* include GP visits; other health practitioner consultations such as counseling services, dieticians, and physiotherapists; emergency department visits; hospital inpatient stays in acute or mental health settings; mental health outpatient visits; and medication use.
3. *Personal and family costs* include the use of alternative therapies such as massage and reflexology, as well as the use of health promotion resources such as sporting facilities and support groups. Travel costs to attend health services and health promotion activities will also be included. The time cost for using the mobile website will not be included as this will occur during leisure time and is expected to be minimal.
4. *Productivity costs* include time spent absent from work owing to illness.

#### Measurement of Costs and Benefits

Benefits will be measured using 5-dimension EuroQoL. Intervention costs will be measured through discussions with the mobile website developers about the requirements to maintain the website for a 1-year period. A plan for promoting the mobile website to future ICU survivors will be prepared, detailing the elements that will be included. Health care costs will be measured by a patient diary for GP visits, health practitioner visits, and medication use. Linked data will be used to measure emergency department visits, outpatient visits, and hospital usage. Personal and productivity costs will be measured by a patient diary, which will record the number of times health-related services and health promotion resources are accessed, travel details to access these resources, and time spent absent from paid work.

#### Valuation of Costs and Benefits

Benefits will be valued by calculating quality adjusted life years from the 5-dimension EuroQoL data using Australian derived utility weights [21]. Intervention costs will be valued by obtaining 3 quotes from service providers. The average of the 3 quotes will be used in the calculations. Health care costs will be valued using the Australian Medicare Benefits Schedule [22], Pharmaceutical Benefits Scheme data [23], and the National Hospital Cost Data Collection [24]. Personal and family costs will be valued by cost prices recorded in the patient diaries. Travel costs will be valued by the number of visits to providers or facilities, the average distance traveled, and the Australian Taxation Office guidelines for car expenses (cents per kilometer method) [25] or the cost of public transport. Productivity costs for time spent absent from paid work will be valued using the human capital approach [26].

#### Data and Sensitivity Analysis

The incremental cost effectiveness ratio will be calculated by dividing the difference in costs between the intervention and control arms by the difference in quality-adjusted life years.

Probabilistic sensitivity analysis using Monte Carlo simulations will be performed. Uncertainty exists around the estimate of the intervention effect on quality of life and around costs, including the health provider, individual, and productivity costs, and these will be included in the sensitivity analysis with the distributions to be determined from the data. Results will be presented as scatterplots and cost effectiveness acceptability curves for a range of willingness to pay thresholds.

No discounting is required because all costs and benefits will be measured within a 1-year time period. All costs will be valued using Aus $ 2018. No modeling of costs and benefits into the future will be undertaken.

## Results

Participant recruitment is currently underway. Recruitment is anticipated to be completed by December 2020 and the first results are expected to be submitted for publication in 2021.

## Discussion

ICU survivors face many challenges during their recovery, and many may never achieve a level of recovery that is acceptable to them. Despite the evidence confirming that these individuals have an increased uptake of health care resources and poor outcomes, they are not routinely offered dedicated programs to support them post discharge from ICU.

This research study is an innovative approach to providing an evidence-based recovery program for ICU survivors, a group of individuals who experience significant levels of physical and psychological morbidity. The findings from this study will inform a larger study and wider debate on approaches to engage and support survivors post ICU.

## Abbreviations

GP: general practitioner
HADS: Hospital Anxiety and Depression Scale
ICU: intensive care unit
